# Sustainability on Bread: How Fiber-Rich Currant Pomace Affects Rheological and Sensory Properties of Sweet Fat-Based Spreads

**DOI:** 10.3390/foods12061315

**Published:** 2023-03-20

**Authors:** Anne-Marie Reißner, Harald Rohm, Susanne Struck

**Affiliations:** Chair of Food Engineering, Institute of Natural Materials Technology, Technische Universität Dresden, 01062 Dresden, Germany

**Keywords:** currant pomace, dietary fiber, berry juice processing, by-product, rheology, texture, sensory perception, planetary ball milling

## Abstract

Dietary fiber may contribute to increasing the nutritional value of “unhealthy food”—for instance, spreads with high fat and sugar content. The high amount of fiber and the presence of phenolic compounds, organic fruit acids, and aroma compounds make currant pomace a promising ingredient to be used in a wide range of foods. However, the particle size of this by-product is a key factor influencing texture, rheology, physical stability, and sensory properties of the final commodities. Wet planetary ball milling of seedless currant pomace suspended in oil resulted in particles <30 µm, which is required for a creamy texture. Spread stiffness and viscosity were adapted by lowering the solid fat content in a way that the fruity spreads with 16 g/100 g pomace resembled a sweet commercial nut spread. The pomace showed stabilizing effects, as oil separation was reduced and a viscosity increase during storage was prevented. Principal component analysis after sensory flash profiling of five formulations highlighted differences in fruitiness, sweetness, greasiness, and viscosity. Hence, depending on the pomace level and fat composition in the formulation, the properties of sweet spreads can be specifically designed to fulfill the respective requirements. Additionally, sweet and savory wafer fillings provide great potential to be enriched with fruit pomace.

## 1. Introduction

Sweet spreads mainly comprise vegetable fat (20–46 g/100 g); sugar (41–56 g/100 g); and flavoring components (3–16 g/100 g), such as cocoa, milk powder, and/or vanilla [1,2,3]. Increasing the nutritional value of such food typically described as “unhealthy” is desirable to expand the target consumer group. In this particular context, dietary fiber can be used to substitute fat or sugar in such a complex system. The substitution of dietary fiber may contribute to enhancing the still-too-low fiber intake which, depending on the source, should be approx. 8–20 g/1000 kcal energy intake [4]. According to European regulations, products including at least 3 g/100 g dietary fiber or 1.5 g fiber per 100 kcal can be labeled as being a “source of fiber”, and products containing above 6 g/100 g (3 g/100 kcal) as “high in dietary fiber” [5]. A plant-based hazelnut spread with 3 g/100 g soybean fiber and a total dietary fiber content of 6.8 g/100 g is an already commercially available example [6].

In his recent overview on cream-filling formulations, Tiefenbacher [3] suggested replacing sugar with short smooth fiber—for instance, 6.35 g/100 g pea fiber or 8 g/100 g inulin, and using fruit powders or citrus oil instead of artificial flavors to mask potential flavor issues. Other authors investigated the effect of incorporating chestnut flour (up to 10 g/100 g) on texture, rheology, and sensory attributes of fat-based spreads [1] or substituting 20 g/100 g cocoa partially or completely for carob pod and chicory root powder [7], mainly aiming at reducing the spreads’ caffeine content. However, commercially available reference products were not included in these studies, making it difficult to interpret the observed impact on the rheological properties of the spreads. Phoon and Henry [8] reported that oleogels, which were produced by freeze-drying fiber containing oil-in-water emulsions, were stable without showing oiling-off after compression, and that it was possible to mimic the spreadability of target products, such as soft breakfast spreads (for instance, Nutella^®^ from Ferrero International S.A., Alba, Italy) or bakery margarine by this approach.

Currant pomace is an underestimated by-product of juice production, rich in dietary fiber and polyphenols [9]. Several studies showed its high potential when used in cereal-based foods [10,11,12,13,14,15,16]. In bread, for instance, its high degree of water-binding results in reduced dough stickiness, decreased baking loss, and increased crumb moisture, which is beneficial in terms of product handling, yield, and staling [13]. In fat-based systems, characteristics such as physical stability and particle size become increasingly demanding. Grape pomace (<200 µm) in chocolate spread negatively affected its consistency and smoothness, increased firmness, and introduced a foreign taste, but the total phenolic content increased when sugar and milk components were substituted up to 15 g/100 g [17]. The authors highlighted its potential as a low-cost bulking agent but stressed the need for optimized processing with respect to particle characteristics. Fiber microparticles (<10 µm) produced by spray-drying could be promising in the replacement of fat in hazelnut spreads while maintaining spreadability, a creamy texture, sweetness, and flavor [18]. 

Recently, we micronized seedless currant pomace in a planetary ball mill, aiming at modifying its technofunctional properties [19]. After 2 h of dry-milling, the powder was characterized by an intensified reddish color, an increased swelling capacity, and a volume-based median of 7 µm (*x*_90_ = 22.5 µm). The total dietary fiber content of the seedless pomace was 71.3 g/100 g (5.6 g soluble fiber, 65.7 g insoluble fiber) and not affected by processing.

In this study, sweet spread formulations with seedless currant pomace (SCP) were designed to gain knowledge on specific processing requirements and resulting product characteristics. When pomace is added to such a complex multiphase system with different solid particles dispersed in a continuous oil phase, textural characteristics may change. However, the spread should neither damage the crumb (when being too firm) nor run off a slice of bread. When using entire SCP instead of isolated fiber, advantages in flavor may also be taken from its aroma profile [15].

## 2. Materials and Methods

### 2.1. Pomace Processing

Blackcurrant and redcurrant pomace were provided by Austria Juice GmbH (Allhartsberg, Austria). Before separating the juice in hydraulic belt presses, pectinase was added to the mashed berries. The residual pomace was then dried at 75 °C for 6 h in an H01 compact cabinet dryer (Harter GmbH, Stiefenhofen, Germany). By means of a GM200 knife mill (Retsch GmbH, Haan, Germany), the dried pomace was comminuted for 60 s at 5000 rpm, ensuring that the seeds were not crushed. The seedless fiber-rich fraction containing skins, pulp, and stems was subsequently separated by sieving through a 0.6 mm sieve for 2 min at an amplitude of 1.4 mm (AS200, Retsch GmbH, Haan, Germany). 

The obtained seedless pomace was further processed by planetary ball milling to reduce particle size. The 250 mL grinding jars of the PM400 (Retsch GmbH, Haan, Germany) were each loaded with a bulk of approx. 150 mL steel balls (d = 3 mm) and 50 mL bulk volume of SCP dispersed in 90 mL canola oil. Because of a bulk density of 0.56 g/mL, this amount refers to 28 g SCP. Wet-milling was then conducted at 300 rpm with direction reversal every minute for 100 min in duplicate [19]. Samples were taken in intervals of 20 min.

The particle size distributions of dried SCP and suspensions of SCP in oil were determined with a HELOS/KR-H2487 laser diffraction spectrometer (Sympatec GmbH, Clausthal-Zellerfeld, Germany). Prior to analysis, the suspensions were dispersed in ethanol to reach an optical density of 20–35% (Cuvette 50, Sympatec GmbH). Powders were dispersed at 0.3 MPa after passing through a 2000 μm sieve. Volume-based median value x_50_, x_90_, and x_99_ (50%, 90%, and 99% of particles smaller than this size, respectively); Sauter mean diameter (SMD); and the specific surface area referring to the monomodal spherical particles of SMD were calculated from the size distribution densities with the PAQXOS 4.3 evaluation software.

### 2.2. Preparation of Sweet Spreads

For incorporation in sweet spreads, SCP was wet-milled for 80 min. Instead of pure canola oil, the continuous phase consisted of canola oil (Brökelmann + Co − Oelmühle GmbH + Co, Hamm, Germany) and palm fat (Bavettin 2280, Walter Rau Neusser Öl und Fett AG, Neuss, Germany) in different ratios, lecithin (F600 IPM, Lecico GmbH, Hamburg, Germany), and vanillin (Heinrich Karow Nachf. KG, Plauen, Germany). Prior to milling, the components were mixed at 50 °C for 30 s with an MQ955PE handheld blender (Siemens AG Österreich, Wien, Austria). To finalize the spreads, icing sugar (Ultrafine S00, Couplet Sugars, Brunehaut-Wez, Belgium) with an average particle size of 11 μm (x_99_ = 28 μm) was added to the milled pomace-in-oil suspensions and equilibrated at 60 °C for 1 h using the MQ955PE blender. Analyses were conducted after conditioning the spreads for 24 h at 25 °C. Viscosity and oil separation were also measured after storing the spreads for 23 d at 25 °C.

**Figure 1:**
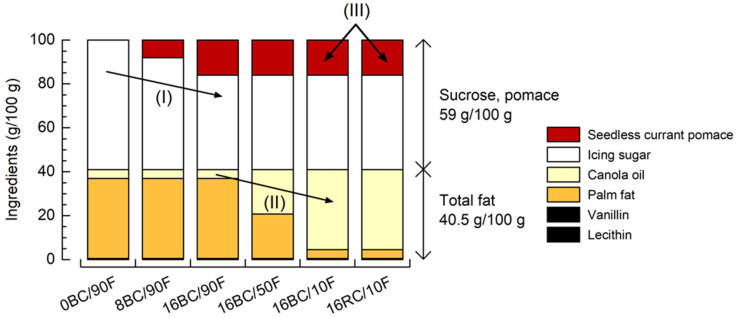
Formulations of sweet spreads with different amounts of seedless blackcurrant pomace powder (I), different fat compositions (II), and redcurrant pomace powder (III). BC—blackcurrant; RC—redcurrant; F—palm fat. Coding refers to pomace content (g/100 g) and ratio of palm fat related to total fat (%).

We investigated six formulations of sweet spreads (Figure 1), which varied in pomace content (I), fat composition (II), and pomace variety (III). Starting with a pomace-free white reference (0BC/90F) and based on preliminary experiments, the content of blackcurrant (BC) was set to 8 (8BC/90F) or 16 g/100 g (16BC/90F) by replacing the same amount of sugar (I). Total fat content was similar in all spreads (40.5 g/100 g), but the ratio of palm fat to canola oil depended on the respective formulation. At a level of 16 g/100 g pomace, the fraction of palm fat was set to 90%, 50% (16BC/50F), or 10% (10BC/10F) (II). Finally, an additional formulation was prepared using seedless redcurrant pomace powder (16RC/10F) instead of blackcurrant pomace powder (III).

### 2.3. Spread Analysis

Rheological measurements were performed on two individually prepared spreads, with 5.5 g sample material placed on a Peltier-controlled lower plate of a Physica MCR 300 rheometer (Anton Paar Germany GmbH, Ostfildern-Scharnhausen, Germany) adjusted to 25 °C. The gap between the lower plate and the 40 mm diameter serrated upper plate was set to 1 mm [20]. After 10 min resting period, a frequency sweep from 0.25 to 100 rad/s was performed in the linear viscoelastic region at a strain of 0.3% (10 points per decade). The apparent viscosity of spreads at 25 °C (typical consumption temperature) and 50 °C (processing temperature) at a shear rate of 10 1/s were derived from flow curves with logarithmically increasing shear rate from 0.001 to 100 1/s with 10 points per decade and time setting per measurement point between 100 s and 2 s.

Phase separation at accelerated conditions was measured in duplicate on two independent spreads (*n* = 4). An amount of 7.5 ± 0.1 mg of fresh or stored spread (see Figure 1) was centrifuged at 20,000× g and 25 °C for 10 min, and the separated oil was removed using a pipette. The oil separation (g/100 g) was calculated as oil released from the initial mass [1]. The rheological and structural properties were also examined for the commercially available hazelnut–cocoa spread Nutella^®^ (Ferrero Deutschland GmbH, Frankfurt, Germany).

A flash profile analysis following DIN standard 13299 [21] on the pomace-containing spreads was carried out in two sessions with ten trained assessors with university background, profound sensory education, and regular participation in sensory studies. Prior to participation, all subjects gave informed consent and agreed to the study conditions. Two spoons with 3 g sample each were served on encoded Petri dishes in individual sensory booths of a sensory laboratory. Water and wheat bread were served to neutralize taste. In the first session, the respondents were asked to identify their individual descriptors for attributes related to smell, taste, and mouthfeel for all pomace-containing spreads. After a break of at least 1 h, the assessors were then asked to use their individual attributes to rate the spreads using unstructured scales of 10 cm length. Each of the scales with the respective attributes was labeled with “imperceptible” on the left and “pronounced” on the right.

### 2.4. Statistics

Analyses of variance with subsequent Student–Newman–Keuls post hoc testing at *p* ≤ 0.05 were conducted with SAS^®^ OnDemand for Academics (SAS Institute Inc., Cary, NC, USA). A biplot was obtained after generalized Procrustes analysis on data of flash profiling with Senstools (OP & P Product Research BV, Ede, The Netherlands).

## 3. Results and Discussion

### 3.1. Particle Size Reduction of Seedless Currant Pomace

The main challenge for the use of pomace in fat-based systems is, apart from physical stability, pomace particle size, which should be below approx. 30 μm to ensure a smooth texture and a pleasant mouthfeel [22]. Such small particles could not be obtained by milling pomace with impact mills [9,17] but, after separation of the seeds, it was possible to comminute the remaining fraction by, for instance, dry planetary ball milling or wet-milling in a high-speed circulation mill [19,23]. Table 1. shows the time-dependent decay of particle size distribution parameters of seedless blackcurrant pomace during wet planetary ball milling performed in this study.

The initial particle size median of 256.5 ± 1.5 µm was reduced to 11.7 ± 0.3 µm within the first 20 min. After milling for 80 min, the size of almost all particles (x_99_ = 31.2 ± 1.2 µm) was below 30 μm. Based on these results, suspensions milled for this particular time were chosen for spread processing. The corresponding Sauter mean diameter was 2.8 ± 0.1 µm and the specific surface area calculated from this measure increased from approx. 0.1 m²/mL (initial pomace) to 2.1 ± 0.0 m²/mL. For comparison, dry-milling of SCP for 2 h resulted in x_99_ = 46.7 ± 2.3 μm (SMD = 3.6 µm), and surface area after 4 h dry-milling was 2.5 m²/mL [19]. The increased specific surface area can be considered an indicator for a higher adsorption capacity and a potentially improved bioavailability of nutrients and bioactive compounds [19,24]. The more effective comminution during wet-milling can be attributed to the fact that the use of dispersion medium counteracts agglomeration and also increases friction [25].

### 3.2. Rheological Properties and Physical Stability of Sweet Spreads

**Figure 2:**
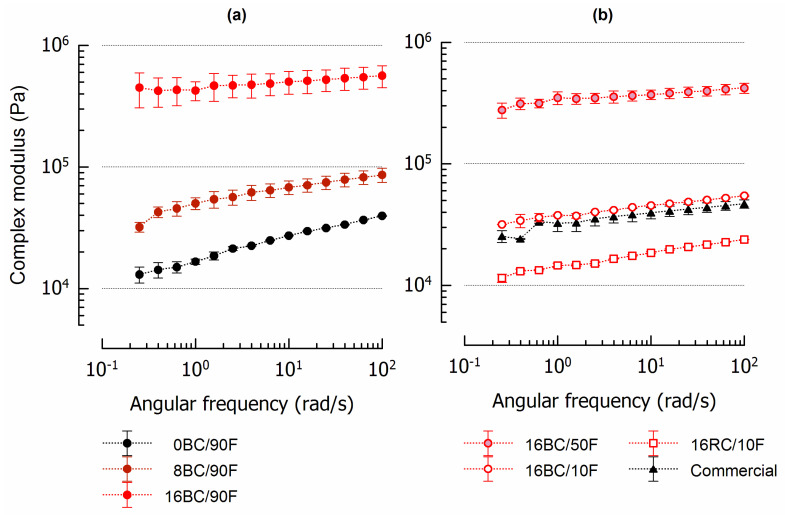
Frequency dependency of sweet spreads at 25 °C: (**a**) Formulations with increasing pomace content; (**b**) Formulations with constant pomace content but varying fat composition plus a commercial nut spread. Coding refers to pomace content (g/100 g), pomace variety (BC—blackcurrant; RC—redcurrant), and the fraction of palm fat (F) in total fat (%). To improve clarity, not all data points are plotted.

Figure 2 shows the complex modulus G* as a measure of stiffness of six spread formulations varying in pomace content, the pomace variety included, and fat composition. In addition, the complex modulus of a sweet commercial nut spread is also shown. By raising the pomace content in steps of 8 g/100 g, stiffness increased nonlinearly. Formulation 8BC/90F was up to 2.6 times stiffer than the pomace-free reference (0BC/90F), whereas 16BC/90F was 6.5–12 times stiffer (lowest and highest frequency) than 8BC/90F (Figure 2a). The pomace-containing spreads were less dependent on frequency *ω* than 0BC/90F. The included pomace increased elastic contributions, indicated by the loss tangent, which was, on average for 1 < *ω* < 100 rad/s, highest for 0BC/90F (0.293 ± 0.010), and lowest for 16BC/90F (0.086 ± 0.006) and 16BC/50F (0.081 ± 0.006). Acan et al. [17] substituted sugar and milk components in a chocolate–nut spread with up to 15 g/100 g grape pomace, using an experimental design approach. They also observed increased stiffness and higher elastic contributions in spreads containing pomace, but the complex interactions between the ingredients in the 14 formulations limited building a model for consistency prediction.

When pomace content was kept constant but with palm fat partially replaced by canola oil, G* decreased by about 1.3 × 10^5^ Pa in case of 16BC/50F and by an order of magnitude for 16BC/10F (Figure 2b). The rheological properties of 16BC/10F at small deformation were almost similar to those of the commercial spread, which was also reflected by the loss tangent (16BC/10F: 0.137 ± 0.007; commercial spread: 0.135 ± 0.009). The formulation containing redcurrant instead of blackcurrant was less stiff, likely because 16RC/10F, despite identical milling conditions, contained coarser particles (x_90_ = 43.8 ± 4.7 µm) than the other spreads (e.g., 16BC/10F x_90_ = 25.1 ± 0.2 µm). Larger particles exhibit a smaller surface area, are less porous, and generally immobilize less oil than fine particles [26]. After rheological analyses of nut spreads varying in solid fat content, Glicerina et al. [20] summarized that elastic structures formed through interactions among fat crystals trap the liquid oil. They also found nonlinear leaps in the storage modulus (*G*’) between the formulations, which were designed in equal intervals. Two commercial Italian nut spreads were characterized by a *G*’ at *ω* = 10 rad/s of approx. 2 × 10^4^ Pa and 8 × 10^4^ Pa. The pomace-containing samples 8BC/90F and 16BC/10F as well as the reference (0BC/90F) of our study fit excellently into this range. 

**Figure 3:**
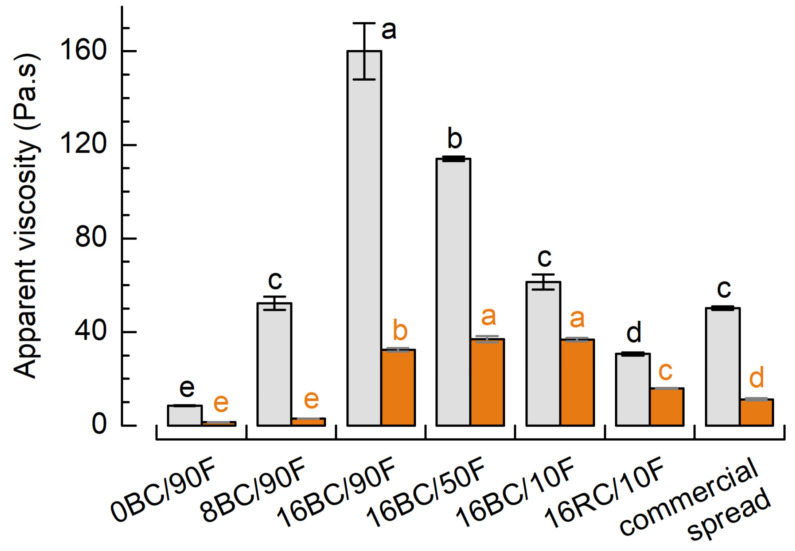
Apparent viscosity (at a shear rate of 10/s) of sweet spreads at 25 °C (grey bars) and at 50 °C (orange bars). Coding refers to pomace content (g/100 g), pomace variety (BC—blackcurrant; RC—redcurrant), and the ratio of palm fat (F) related to total fat (%) (see Figure 1). Different superscripts indicate significant differences (*p* < 0.05) between viscosity at the respective temperature.

As indicated by the flow curves from rotational shear experiments, all spreads showed shear thinning behavior (data not shown). The highest apparent viscosity (at a shear rate of 10/s) was observed for 16BC/90F (160.0 ± 12.0 Pa.s), the lowest viscosity for 0BC/90F (8.6 ± 0.2 Pa.s) (Figure 3). Just as observed in small deformation oscillating measurements, the viscosity of 8BC/90F (52.3 ± 2.8 Pa.s) and 16BC/10F (61.4 ± 3.2 Pa.s) was also most similar to that of the commercial spread included in this study (50.2 ± 0.7 Pa.s). Vegetable oil spreads made after mixing oleogels, hydrogels, and finally containing 15 g lingonberry pomace (particle size below 500 µm) showed a similar viscosity (~60 Pa.s) at 10/s [27]. In addition, images showing these pomace spreads on bread characterized them as being easily spreadable. 

At a typical processing temperature of 50 °C, the impact of the pomace on viscosity is evident. When the fat was completely melted, the formulations with 16 g/100 g pomace showed the highest viscosities (16–37 Pa.s). Although 16RC/10F appeared at room temperature to be softer than the commercial spread, its viscosity at 50 °C was significantly higher, whereas 8BC/90F showed reduced viscosity as compared to the commercial spread. When it comes to determining processing conditions such as pumping or dosing, those effects become important and must be considered. 

**Figure 4:**
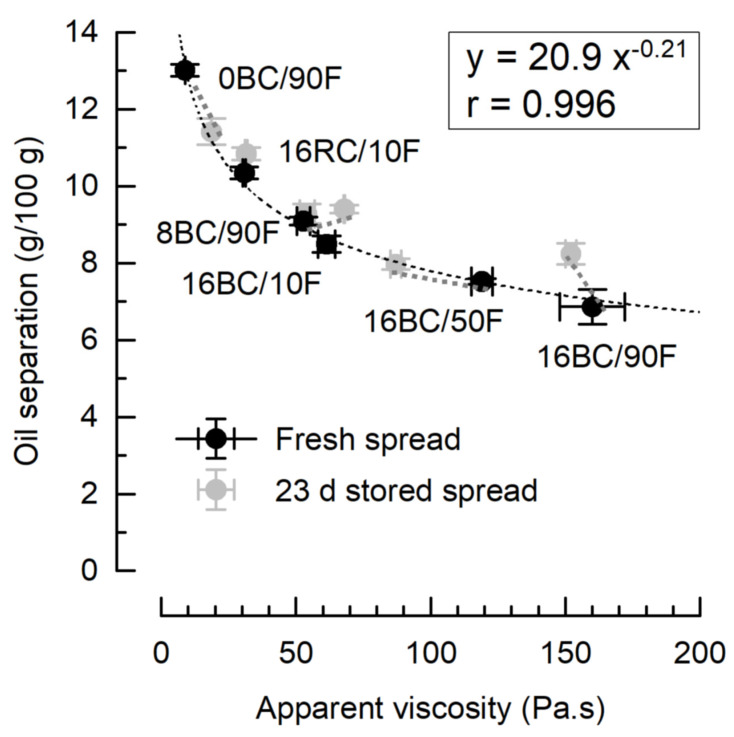
Oil separation at 25 °C and apparent viscosity (at 10/s) of sweet spreads as affected by storage. Coding refers to pomace content (g/100 g), pomace variety (BC—blackcurrant; RC—redcurrant), and the ratio of palm fat (F) related to total fat (%).

Regarding physical stability, oil separation was highest for 0BC/90F (13.0 ± 0.1 g/100 g), and significantly lower in formulations that contained pomace because of its oil binding capacity (Figure 4) [19]. Oil separation of 16BC/90F (6.9 ± 0.5 g/100 g) did not differ significantly (*p* > 0.05) from that of the commercial spread (7.1 ± 0.1 g/100 g). Susceptibility for oil separation was also low in the other formulations with 16 g/100 g blackcurrant pomace that contained less solid palm fat (16BC/10F and 16BC/50F), but this effect was smaller than the contribution of the pomace.

Overall, we observed a nonlinear correlation (r = 0.996) between oil separation and apparent viscosity, best described by the power function y = 20.9 x^−0.21^. When viscosity is high, less oil is transported to the spread surface [28]. As oil separation in nut spreads is an ongoing issue that affects quality and appearance [29], fiber-rich fruit pomace may decelerate oil migration. Doing so, its polyphenols may also have a positive impact on oxidative stability. After 30 d of storage at 37 °C (accelerated conditions), peroxide values of vegetable oil spreads with lingonberry pomace were significantly lower than the pomace-free references [27]. The authors linked this to the natural presence of phenolic compounds, which are also abundant in currant pomace. 

After storing the spreads for 23 d at 25 °C, viscosity of the reference 0BC/90F almost doubled, which might have caused the time-based reduction of oil separation. Presumably, the fat recrystallized from meta-stable to energetically more favorable and larger β-crystals [30]. In contrast, pomace at levels of 16 g/100 g prevented the increase in viscosity, probably due to steric effects, and 16BC/50F even showed reduced viscosity. This might be the main reason why the oil separation increased for pomace spreads slightly but significantly after storage. However, the relation of samples to each other remained basically constant, and it can be concluded that pomace enhanced the physical stability of the spreads.

### 3.3. Sensory Attributes of Pomace Spreads

Fruit pomace is expected to change the sensory profile of foods and, depending on substitution level and product, this effect may raise or decrease acceptability. In bread, blackcurrant pomace introduced more than one hundred volatiles [15]. Irrespective of textural changes, approximately 10% fruit pomace (e.g., apple or grape) increased affective hedonic ratings for sweet and savory cereal products such as cakes, cookies, and extruded snacks [31,32,33]. In addition, the impact of chokeberry and tomato pomace on the flavor of tea and jam was exploited [34]. 

**Figure 5:**
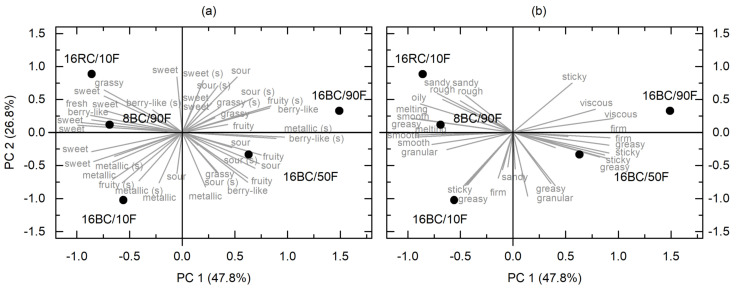
Biplots describing sensory differences between sweet spreads containing currant pomace: (**a**) Loadings with flavor attributes ((s) refers to smell); (**b**) Loadings with texture/mouthfeel attributes. Spread coding refers to pomace content (g/100 g), pomace variety (BC—blackcurrant; RC—redcurrant), and the ratio of palm fat (F) related to total fat (%). Only loadings, which were mentioned repeatedly, are labeled.

In this study, we use a flash profiling procedure to obtain flavor attributes typical for the pomace and to highlight perceived differences between the spreads with different types and/or amounts of pomace and different fat compositions. However, this method may ignore flavor attributes, which are characteristic of currant pomace, as long as differences in these characteristics are not clearly perceptible. Figure 5 shows the five pomace-containing spreads in biplots with loadings representing flavor texture attributes.

After generalized Procrustes analysis, three factors with eigenvalues >1 were extracted and accounted for 90.4% of the total variance. Figure 5 displays principal component 1 (PC 1) versus PC 2, both accounting for 74.6% of the total variance. A high confidentiality in the real data set (82.8%) was obtained, which was higher than the upper 5% of the total variance accounted for in the permutated data sets. As the impact of PC 1 is higher than that of PC 2, samples in the x-direction differed more among each other than samples in the y-direction. Hence, 16BC/90F deviated most noticeably from the spreads with negative PC 1 coordinates. Corresponding attributes, which loaded especially in the positive direction of PC 1, were “sticky”, “firm”, and “viscous” regarding texture and “sour”, “fruity”, and “grassy” regarding flavor. “Sweet” was chosen by nine of ten assessors to discriminate the spreads and loaded off from “sour” predominantly in the upper left segment (negative PC 1; positive PC 2). Indeed, 8BC/90F contained more sugar than the other spreads, but concurrently, 16RC/10F—the spread with redcurrant—was perceived as extra sweet. Both metallic smell and taste were repeatedly mentioned for 16BC/10F. Apparently, this attribute became more pronounced when the ratio of oil was high in spreads with blackcurrant pomace.

Overall, the sensory-perceived texture attributes support the findings of rheological and particle size measurements: 16RC/10F, which contained larger particles than blackcurrant spreads, was characterized as “sandy” but also as “oily”, whereas formulations with more palm fat showed increased viscosity. Although the findings allow no conclusions on consumer acceptability, they stress that particles below 30 μm are essential for a smooth mouthfeel. By means of dry or wet ball milling, this demand can be fulfilled. The spread 16BC/10F was, from the rheological point of view, most similar to a popular commercial spread and may be accepted by a wide audience for this purpose, whereas firmer spreads such as 16BC/90F may fit better for applications such as wafer fillings, as currant pomace had stabilizing effects on texture and decreased the oil separation.

## Figures and Tables

**Table 1:** Effect of wet-milling time on particle size measures of seedless blackcurrant pomace in canola oil.

Milling Time (min)	*x*_50_ (µm)	*x*_90_ (µm)	*x*_99_ (µm)
0 (starting point)	256.5 ± 2.1	532.2 ± 16.9	701.2 ± 8.4
20	011.7 ± 0.3 ^a^	042.7 ± 2.1 ^a^	073.1 ± 2.9 ^a^
40	007.9 ± 0.2 ^b^	028.0 ± 0.3 ^b^	053.9 ± 1.7 ^b^
60	005.6 ± 0.1 ^c^	017.2 ± 0.6 ^c^	035.4 ± 1.3 ^c^
80	004.6 ± 0.1 ^d^	014.4 ± 0.8 ^cd^	031.2 ± 1.2 ^cd^
100	004.1 ± 0.1 ^d^	012.0 ± 0.4 ^d^	025.7 ± 1.2 ^d^

Mean values (± half deviation range) in a column with different letters differ significantly (*p* < 0.05).

## Data Availability

The data that support the conclusions of this study are available upon reasonable request from the corresponding author.
